# Management of deltopectoral flap failure using a three-stage revision reconstruction: a case report

**DOI:** 10.1093/jscr/rjab143

**Published:** 2021-04-24

**Authors:** Adit Chotipanich, Sombat Wongmanee

**Affiliations:** Head and Neck Unit, Department of Medical Services, Chonburi Cancer Hospital, Ministry of Public Health, Chonburi, Thailand; Head and Neck Unit, Department of Medical Services, Chonburi Cancer Hospital, Ministry of Public Health, Chonburi, Thailand

## Abstract

Although deltopectoral flap failure is uncommon, its management can be difficult. This report presents a case of deltopectoral flap failure successfully rescued by a three-stage revision reconstruction using the postdebridement flap. A 59-year-old patient presented with a pharyngocutaneous fistula due to radionecrosis and subsequently underwent a medially based deltopectoral flap reconstruction for fistula closure. Unfortunately, this operation was unsuccessful because the flap developed necrosis at its distal tip, and the postdebridement flap could not be directly placed on the defect because of its shorter length. A subsequent revision operation successfully closed the fistula using a three-stage reconstruction with the postdebridement flap. Although this three-stage technique can avoid the morbidity associated with additional flap harvesting and can greatly extend the distance to the recipient, it also requires more time to heal and more operations than simply harvesting a new flap.

## INTRODUCTION

Despite the advent of microsurgery, creation of a deltopectoral flap is still useful in some clinical scenarios. This type of flap is most appropriate in patients who require a reconstruction of the lower face or neck regions, but who are not ideal candidates for a free flap reconstruction [1]. One disadvantage of this technique is the need to process the pedicle 2 or 3 weeks after the primary surgery for flaps requiring a two-stage design. The blood supply of a deltopectoral flap is usually provided primarily by the second, third and fourth intercostal perforating vessels [2].

Deltopectoral flap failure is not common; however, it can occur if there is an inadequate blood supply to the flap’s distal end, especially when a longer flap is required [3]. When failure occurs, the resulting necrotic portion must be removed. Because the postdebridement flap length is shorter, it may not have an adequate length to reach the defect without tension, forcing surgeons to completely discard the partially failed flap.

This report presents the case of a patient who developed deltopectoral flap failure after a pharyngocutaneous fistula closure operation, which was successfully rescued by a three-stage revision reconstruction using the postdebridement flap.

## CASE REPORT

**Figure 1:**
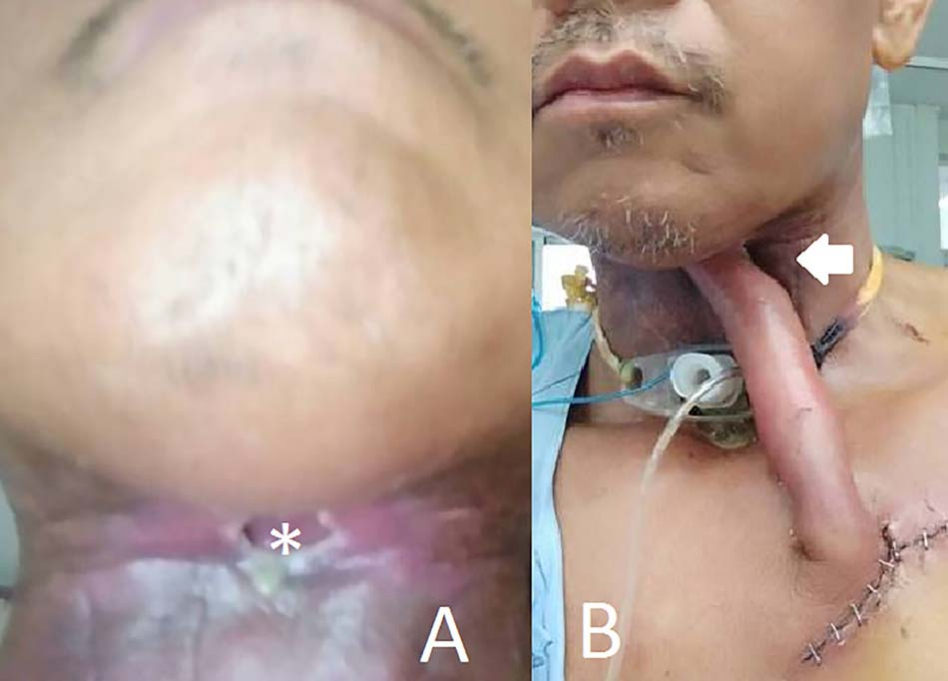
(**A**) Shows the preoperative fistula with a diameter of 2 cm and (**B**) shows the patient on the fifth day after the first surgery.

A 59-year-old Thai man was successfully treated with chemoradiation for stage III glottic cancer 3 years prior to presentation. Unfortunately, the tumor recurred and the patient was sent to our clinic for salvage surgery involving a total laryngectomy. Two months after this salvage surgery, the patient developed radionecrosis of the soft tissue of the neck. This necrosis resolved after hyperbaric oxygen treatment (HBOT); however, a large pharyngocutaneous fistula remained in the upper neck (Fig. 1A).

A free flap reconstruction was not suitable for this patient because of his surgical history and severe neck fibrosis. Thus, a two stage, medially based deltopectoral flap reconstruction was chosen. Because the distance requirement was relatively long, we opted for a surgical delay for the distal two-thirds of the flap. The flap was completely elevated and placed on the fistula 10 days after this delay.

On postoperative day 5, the flap developed necrosis at its distal tip and was completely separated from the fistula (Fig. 1B). The flap tip was trimmed to ~2 cm; however, this postdebridement flap did not have a sufficient length to reach the fistula. After discussion with the patient, we decided to re-operate using this shortened flap rather than harvesting a new flap from the contralateral chest.

**Figure 2:**
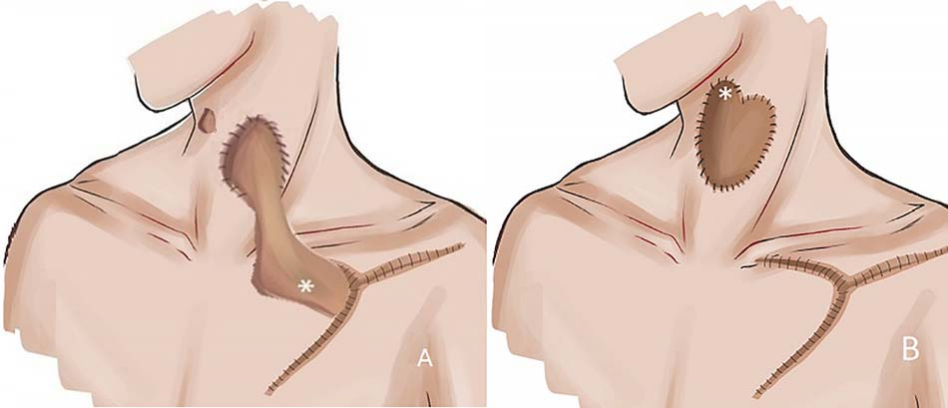
(**A**) Shows a schematic illustration of the first stage of the revision operation. The shortened deltopectoral flap was placed at a temporary recipient site. (**B**) Shows a schematic illustration of the second stage of the revision operation.

The revision operation was divided into three stages. In the first stage, we created a temporary recipient site just below and lateral to the fistula. After debridement, the distal part of the flap was placed on this temporary recipient site (Fig. 2A). After this stage, the patient underwent another course of HBOT.

Because the temporary recipient site was located in a previously irradiated area, the second stage was delayed for 8 weeks, at which point the flap was divided from its origin. The proximal part, which had a reverse blood flow from the temporary recipient site, was transposed upwards to the fistula (Fig. 2B). The patient then underwent a third course of HBOT.

**Figure 3:**
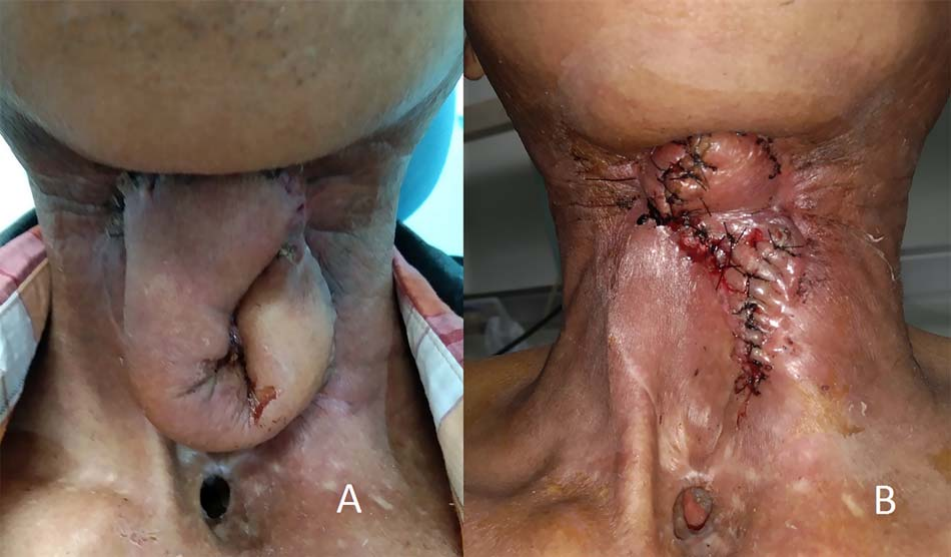
(**A**) Shows the patient at 6 weeks after the second stage of the revision operation, at which point the fistula was completely sealed; (**B**) shows the patient after the third stage of the revision operation.

Approximately 6 weeks after the second stage of the operation, the wound was completely healed without saliva leakage (Fig. 3A). During the third stage, excess flap tissue was divided and removed (Fig. 3B). No further HBOT was administered. By about 1 month after the operation, the patient had resumed regular oral intake.

## DISCUSSION

Creation of a deltopectoral flap is an extremely reliable method for correcting defects in the lower two-thirds of the neck when a primary transfer without delay can be accomplished. For defects of the upper neck or face, however, a longer flap can be created using a delay technique. Unfortunately, this longer flap design has a higher risk of tip necrosis due to insufficient blood supply [3]. When failure occurs, debridement must be performed, and it is generally not possible to resuture the postdebridement flap directly to the defect because of its shorter length. Thus, the surgeon must discard the partially failed flap and harvest a new flap from another location.

In this report, we have presented a three-stage deltopectoral flap reconstruction technique, which uses the shortened postdebridement flap during the revision operation. This technique avoids the morbidity associated with harvesting another flap. In addition, the distance to the recipient can be greatly extended. This case report demonstrates that it is even feasible to perform this technique in patients with a history of radiotherapy.

This three-step reconstruction technique does, however, have some drawbacks. It requires more stages; thus, the treatment takes a longer period of time. In this patient, we were required to further extend the duration between the stages because the temporary recipient site was located in a previously irradiated area.

In conclusion, a three-stage reconstruction can be used as a rescue technique in patients with partial failure at the distal tip of a deltopectoral flap. However, surgeons must carefully consider the drawbacks of this technique before proceeding.
